# *Cryptococcus neoformans/gattii* and *Histoplasma capsulatum* var. *capsulatum* infections on tissue sections: Diagnostic pitfalls and relevance of an integrated histomolecular diagnosis

**DOI:** 10.1093/mmy/myae126

**Published:** 2024-12-28

**Authors:** Alexis Trecourt, Meja Rabodonirina, Marie Donzel, Emmanuelle Chapey-Picq, Abderrazzak Bentaher, Damien Dupont, Charline Miossec, Florence Persat, Martine Wallon, Jean-Philippe Lemoine, Pauline Tirard-Collet, Aline Baltrès, Alexandre Alanio, Mojgan Devouassoux-Shisheboran, Jean Menotti

**Affiliations:** Service de Pathologie Multi-Site - Site Sud, Centre Hospitalier Lyon Sud, Hospices Civil de Lyons, Lyon, France; Faculté de Médecine Lyon-Sud Charles Mérieux, Université Claude Bernard Lyon 1, UR 3738 – CICLY – Equipe Inflammation et immunité de l′épithélium respiratoire, Lyon, France; Service de Parasitologie et Mycologie Médicale, Institut des Agents Infectieux, Hôpital Croix-Rousse, Hospices Civils de Lyon, Lyon, France; Faculté de Médecine Lyon Sud Charles Mérieux, Université Claude Bernard Lyon-1, Lyon, France; Service de Pathologie Multi-Site - Site Sud, Centre Hospitalier Lyon Sud, Hospices Civil de Lyons, Lyon, France; Centre de Recherche en Cancérologie de Lyon (CRCL), INSERM U1052, CNRS UMR 5286, Faculté de Médecine Lyon Sud, Université Claude Bernard Lyon 1, Lyon, France; Service de Parasitologie et Mycologie Médicale, Institut des Agents Infectieux, Hôpital Croix-Rousse, Hospices Civils de Lyon, Lyon, France; Faculté de Médecine Lyon Sud Charles Mérieux, Université Claude Bernard Lyon-1, Lyon, France; Faculté de Médecine Lyon-Sud Charles Mérieux, Université Claude Bernard Lyon 1, UR 3738 – CICLY – Equipe Inflammation et immunité de l′épithélium respiratoire, Lyon, France; Service de Parasitologie et Mycologie Médicale, Institut des Agents Infectieux, Hôpital Croix-Rousse, Hospices Civils de Lyon, Lyon, France; Faculté de Médecine Lyon Est, Université Claude Bernard Lyon-1, Lyon, France; Service de Parasitologie et Mycologie Médicale, Institut des Agents Infectieux, Hôpital Croix-Rousse, Hospices Civils de Lyon, Lyon, France; Faculté de Médecine Lyon-Sud Charles Mérieux, Université Claude Bernard Lyon 1, UR 3738 – CICLY – Equipe Inflammation et immunité de l′épithélium respiratoire, Lyon, France; Service de Parasitologie et Mycologie Médicale, Institut des Agents Infectieux, Hôpital Croix-Rousse, Hospices Civils de Lyon, Lyon, France; Faculté de Médecine Lyon Est, Université Claude Bernard Lyon-1, Lyon, France; Service de Parasitologie et Mycologie Médicale, Institut des Agents Infectieux, Hôpital Croix-Rousse, Hospices Civils de Lyon, Lyon, France; Faculté de Médecine Lyon Sud Charles Mérieux, Université Claude Bernard Lyon-1, Lyon, France; Service de Parasitologie et Mycologie Médicale, Institut des Agents Infectieux, Hôpital Croix-Rousse, Hospices Civils de Lyon, Lyon, France; Faculté de Médecine Lyon-Sud Charles Mérieux, Université Claude Bernard Lyon 1, UR 3738 – CICLY – Equipe Inflammation et immunité de l′épithélium respiratoire, Lyon, France; Service de Parasitologie et Mycologie Médicale, Institut des Agents Infectieux, Hôpital Croix-Rousse, Hospices Civils de Lyon, Lyon, France; Faculté de Médecine Lyon Est, Université Claude Bernard Lyon-1, Lyon, France; Génomique épidémiologique des maladies infectieuses (GENEPII), Institut des Agents Infectieux, Hôpital Croix-Rousse, Hospices Civils de Lyon, Lyon, France; Service de Pathologie, Centre Léon Berard, Lyon, France; Groupe Hospitalier Saint-Louis-Lariboisière-Fernand-Widal, Assistance Publique-Hôpitaux de Paris, Paris, France; Molecular Mycology Unit, CNRS UMR2000, Institut Pasteur, Paris, France; Service de Pathologie Multi-Site - Site Sud, Centre Hospitalier Lyon Sud, Hospices Civil de Lyons, Lyon, France; Faculté de Médecine Lyon-Sud Charles Mérieux, Université Claude Bernard Lyon 1, UR 3738 – CICLY – Equipe Inflammation et immunité de l′épithélium respiratoire, Lyon, France; Faculté de Médecine Lyon Est, Université Claude Bernard Lyon-1, Lyon, France; Faculté de Médecine Lyon-Sud Charles Mérieux, Université Claude Bernard Lyon 1, UR 3738 – CICLY – Equipe Inflammation et immunité de l′épithélium respiratoire, Lyon, France; Service de Parasitologie et Mycologie Médicale, Institut des Agents Infectieux, Hôpital Croix-Rousse, Hospices Civils de Lyon, Lyon, France; Faculté de Médecine Lyon Est, Université Claude Bernard Lyon-1, Lyon, France; Génomique épidémiologique des maladies infectieuses (GENEPII), Institut des Agents Infectieux, Hôpital Croix-Rousse, Hospices Civils de Lyon, Lyon, France

**Keywords:** integrated histomolecular diagnosis, histopathology, targeted massive parallel sequencing, Grocott staining, Alcian blue staining

## Abstract

*Cryptococcus neoformans/gattii* and *Histoplasma capsulatum* var. *capsulatum* may present atypical histopathological features inducing diagnostic errors. We aimed to estimate the frequency of these atypical features in formalin-fixed tissue (FT) samples and to assess the relevance of an integrated histomolecular diagnosis using specific *H. capsulatum* PCR and panfungal PCR followed by Sanger sequencing and/or targeted massive parallel sequencing (MPS). A total of 27 FT from 23 patients with a histopathological diagnosis of cryptococcosis (*n* = 16 FT from 13 patients) or histoplasmosis (*n* = 11 FT from 10 patients) were retrospectively included. All FT were consultation cases. Mycological identifications on equivalent fresh tissue were available for 11/23 (47.8%) patients. The expert pathologist review modified the diagnosis suggested by the initial pathologist in 7/27 (25.9%) FT. Fungal morphology and tissue inflammation were compared between both mycoses. The most discriminant atypical criterion was the presence of dented-looking yeasts, observed in 68.75% (11/16) of *C. neoformans/gattii* and none (0/11) of *H. capsulatum* var. *capsulatum* (*P *= .002). For the 12/23 (52.2%) patients without mycological identification on fresh tissue, an integrated histomolecular diagnosis on FT using specific PCR or panfungal PCR followed by Sanger sequencing and/or MPS led to fungal identification in 9/12 (75%) cases; for cryptococcosis, the targeted MPS sensitivity was higher than that of Sanger sequencing (*P *= .041). Thus, because atypical histopathological features may be tricky, integrated histomolecular diagnosis is essential for optimal patient care.

## Introduction

The classical histopathological features of *Cryptococcus neoformans* and *Histoplasma capsulatum* var. *capsulatum* infections are well known.^1^ On tissue sections, *C. neoformans* is classically described as an encapsulated intra- and extracellular round yeast, measuring 5–10 µm, variable in size, with a thick polysaccharide capsule giving a clear space appearance on the hematoxylin–eosin–saffron (HES)-stained slide and an Alcian blue positivity.^1–5^ Conversely, *H. capsulatum* var. *capsulatum* usually shows a nonencapsulated, exclusively intracellular oval yeast, measuring 2–4 µm, homogenous in size.^1,2,6,7^ These yeasts may cause similar deceptive pseudotumor presentations.^8,9^

These pseudotumor presentations at initial clinical and radiological workup could lead clinicians to send all samples to the department of pathology. However, on formalin-fixed and paraffin-embedded tissue samples (FT), standard mycological identification using culture is no longer possible.^2^ Although histopathological criteria often allow to suggest either cryptococcosis or histoplasmosis, the differential diagnosis between both infections is sometimes challenging while the treatment differs.^1,2,7^ Moreover, both pathogens may coexist in the same geographical areas, complicating the interpretation of epidemiological data.^1^ Furthermore, classical histopathological criteria previously cited are not always present, and/or atypical features may be seen. For instance, the capsule of *C. neoformans* is sometimes inconspicuous,^1^ and this yeast may also rarely produce pseudohyphae or microcells on tissue sections.^10,11^ Similarly, the diagnosis of *H. capsulatum* var*. capsulatum* infections may also be tricky (e.g., “protozoa-like” features on the HES slide).^1^ In addition, several yeasts may resemble *C. neoformans* or *H. capsulatum* var. *capsulatum* on tissue sections, complicating the diagnosis.^1^ Yet, a definitive fungal identification is crucial for patient care. To our knowledge, the relevance of an integrated histomolecular approach on FT^2^ to ensure accuracy and safety during the diagnosis of these infections specifically has never been evaluated.

Our main objective was to estimate the frequency of the atypical histopathological features in *C. neoformans* or *H. capsulatum* var. *capsulatum* infections and to investigate the usefulness of these features in differentiating both mycoses on FT. The secondary objective was to assess the relevance of an integrated histomolecular diagnosis, especially when no mycological identification is available on fresh tissue.

## Materials and methods

### Case selection and collection of data

From 2014 to 2024, available FT (slides and paraffin blocks) with a histopathological diagnosis of cryptococcosis or histoplasmosis (excluding *H. capsulatum* var. *duboisii* infections) were retrospectively included. All FT were sent for review to the Mycology or the Pathology Departments of the Hospices Civils de Lyon (M.R. and A.T.). All histopathological diagnoses were performed prior to the present study for routine practice purposes, based on the histopathological features of *C. neoformans/gattii* and *H. capsulatum* var. *capsulatum* on tissue section.^1,2^ As FT were consultation cases, the initial diagnosis suggested by the referring pathologist was either confirmed or modified by the expert infectious disease pathologist/biologist.

Clinical and laboratory data were retrospectively collected. When available, the results of mycological identification were gathered: (i) direct examination, culture followed by mass spectrometry, *H. capsulatum*-specific PCR,^12^ panfungal PCR followed by Sanger sequencing, all performed on fresh tissue; (ii) cryptococcal antigen detection performed on cerebrospinal fluid (CSF) and/or serum; and (iii) *H. capsulatum*-specific real-time PCR, panfungal PCR followed by Sanger sequencing,^2^ panfungal PCR followed by targeted massive parallel sequencing (MPS),^2^ all performed on FT. Of note, when possible, the fungal molecular identification was performed on FT even if it was already available on fresh tissue and/or CSF/serum. All fungal infections were defined as proven according to the European Organization for Research and Treatment of Cancer and the Mycoses Study Group (EORTC/MSG),^13^ using the histopathological criteria (fungi within tissue injuries/invasion).

The FT from patients studied herein has been declared to the French Ministry of Research (biological resource center authorization number: DC2019-3464).

### Study design

After clinical and laboratory data collection, FT were separated into two groups. Group 1: FT for which a mycological identification was available, using at least one mycological test previously cited, either on fresh tissue, and/or on FT, and/or on CSF/serum. Group 2: FT for which no mycological identification was available (negative mycological test on fresh tissue or CSF/serum, and/or negative molecular test on FT, and/or no fresh sample sent to the mycology laboratory, and/or FT left for diagnosis). Among group 2, patients were successfully treated according to the diagnosis suspected on the basis of histopathological data.

### Histopathological analysis

For each patient, HES-, Grocott-, and Alcian blue-stained slides were performed prior to the study. Herein, for the study purpose, all slides were simultaneously reviewed by a pathologist (A.T.) and two mycologists (M.R. and J.M.). The following histopathological features were collected on the HES-stained slides: presence/absence of a granulomatous inflammation, giant cells, caseous necrosis, capsule (not visible/inconspicuous or clearly visible), and “protozoa-like” features (which may suggest *Leishmania* spp. amastigote form on the HES-stained slide [atypical criterion]). On the Grocott-stained slides, the shape of the yeasts (oval, round, and/or dented-looking [atypical criterion]), the homogeneous or variable size of the yeasts, the intra- and/or extracellular location (i.e., either extracellular yeasts within necrosis or extracellular yeasts outside necrosis area and between inflammatory cells), and the presence/absence of pseudohyphae (atypical criterion) were noted. The staining of the capsule/pseudo-capsule was evaluated using the Alcian blue staining (weak or intense), which is a well-established diagnostic argument for the diagnosis of cryptococcosis on tissue section.^1^

For FT of group 1 (with a mycological identification), after slide reviewing, the proportion of each histopathological feature was compared between *C. neoformans/gattii* and *H. capsulatum* var. *capsulatum* infections, as described below (see section “Statistical analysis”). This was done to identify the histopathological features of interest allowing for better differentiation between both mycoses on FT.

For FT of group 2, although no mycological identification was available, histopathological diagnoses had already been suggested prior to the study by the expert pathologist. Thus, we secondarily investigated whether the histopathological features of interest, previously identified for FT of group 1, were concordant with the histopathological diagnosis initially suggested.

### Molecular tests on FT

#### DNA extraction from FT

DNA from FT was extracted using QIAcube connect (Qiagen, Hilden, Germany) and QIAamp DNA FFPE Tissue kit (Qiagen), as previously described.^2^

#### Specific H. capsulatum var. capsulatum real-time PCR

Fungal DNA was amplified by specific real-time PCR using Hc_24_55F/R primers (forward: 5′-CGTACGACATCATATTAAAAATGA-3; reverse: 5′-CTTTCTTTAAGGTAGCCAAAAT-3′; 0.3 m m of each) and probe Hc_21_79P (5′-FAM-TGTAGTGGTGTACAGGTGAGT-BHQ1-3′; 0.1 m m), as previously described.^12,14^

#### Panfungal PCR and Sanger sequencing

Fungal DNA was amplified by panfungal PCR using two different primer pairs (Eurogentec, Seraing, Belgium) targeting the ITS-2 region: ITS-3/ITS-4 (forward: 5′-GCATCGATGAAGAACGCAGC-3′; reverse: 5′-TCCTCCGCTTATTGATATGC-3′) and MITS-2A/MITS-2B primers (forward: 5′-GATGAAGAACGCAGCGAAAT-3; reverse: 5′-ATGCTTAAGTTCAGCGGGTA-3′), as previously described.^2^ FASTA sequences were aligned to reference sequences from the MycoBank database (https://www.mycobank.org/). A 97% similarity threshold was used for fungal identification.^2^

#### Targeted MPS

Targeted MPS was performed as previously described,^2^ using amplicons from panfungal PCR for library construction. The Illumina Microbial Amplicon Prep kit (Illumina, San Diego, CA, USA) was used for the library preparation. The final library was quantified using the Qubit Fluorometer and the Qubit dsDNA High Sensitivity Assay kit (Thermo Fisher Scientific, Waltham, MA, USA). The library was sequenced using a NextSeq 550 (Illumina). After sequencing, to be considered interpretable, samples had to have more than 80% of reads with a quality score ≥30. Reads were processed using the same in-house bioinformatic pipeline and the same quality criteria and database (UNITE database) as previously described.^2^ Molecular identifications had to have a minimal depth of coverage of 30× to be valid.

### Statistical analysis

Dichotomous variables were expressed as percentages and continuous values as medians. For FT of group 1, a Fisher’s exact test was used to compare the proportions of each histopathological feature between both *C. neoformans/gattii* and *H. capsulatum* var. *capsulatum* infections, using GraphPad (https://www.graphpad.com/quickcalcs/contingency1/). A McNemar’s (with correction for discordant pairs <10) test was used to compare the sensitivities of Sanger sequencing and targeted MPS (https://www.omnicalculator.com/statistics/mcnemars-test). A *P* value < .05 was considered statistically significant.

## Results

### Clinical and laboratory data

**Figure 1.**
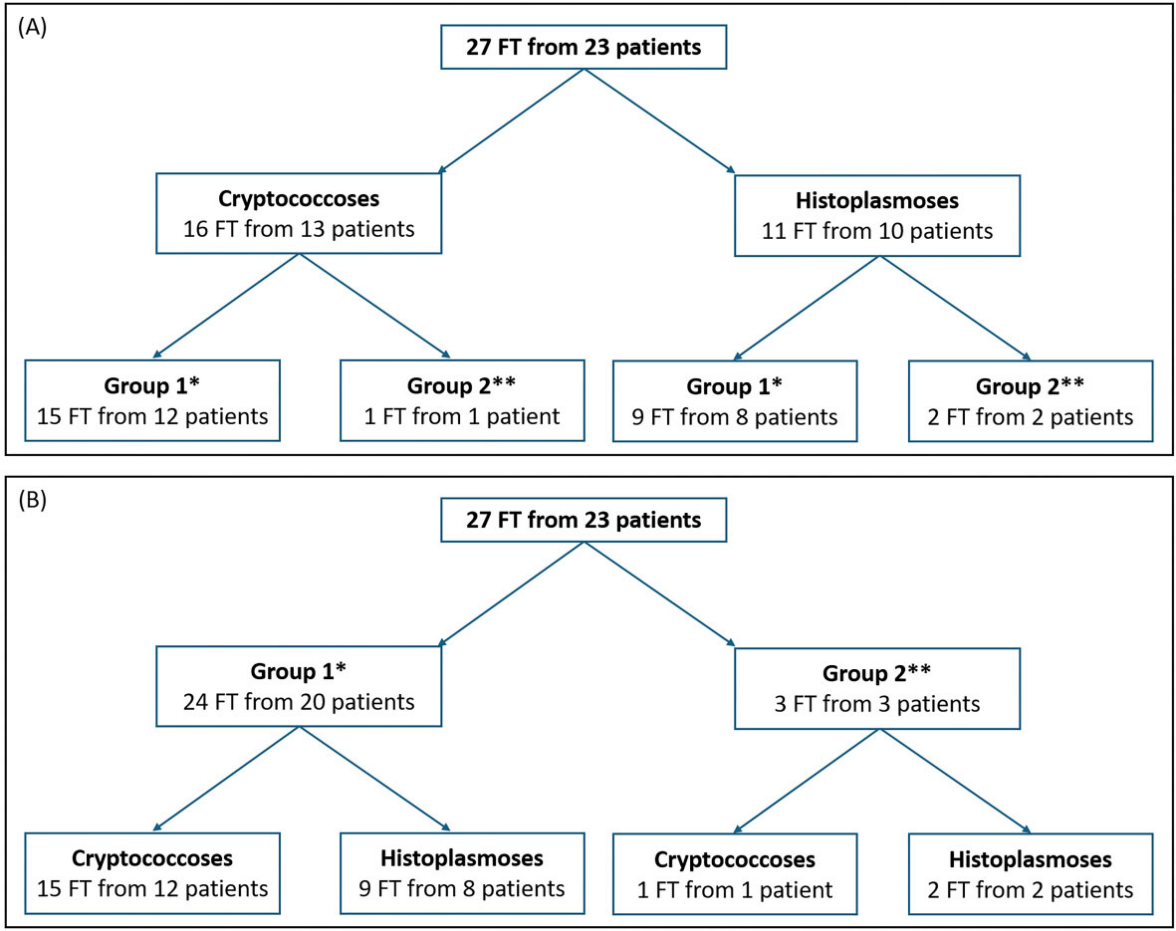
Distribution of patients and FT between mycoses (A) and between groups (B). *Group 1: with mycological identification; **Group 2: without mycological identification. FT: formalin-fixed, paraffin embedded tissues.

A total of 27 FT from 23 patients were retrospectively included. The distribution of patients and FT between groups and mycoses is detailed in Fig. 1. Clinical data and the locations of the biopsies/surgical specimens are detailed in Table 1. The examination of the 27 FT, performed prior to the present study by the expert pathologist, led to the confirmation or modification of the diagnosis suggested by the initial pathologist in 20/27 (74.1%) and 7/27 (25.9%) FT, respectively.

**Table 1.** Clinical data and sampling.

Clinical, laboratory data, and sampling	
**Age at diagnosis** (cryptococcosis, median)	54.1
**Age at diagnosis** (histoplasmosis, median)	54.3
**Male/female ratio** (cryptococcosis)	2.25
**Male/female ratio** (histoplasmosis)	0.67
**Fungal infections** (*n* = 23 patients), % (*n*/*N*)	
Cryptococcosis	56.5 (13/23)
Histoplasmosis	43.5 (10/23)
**Immune status of patients** (*n* = 23 patients), % (*n*/*N*)	
Cryptococcoses (*n* = 13 patients)	
IC patients	84.6 (11/13)
Non-IC patients	15.4 (2/13)
Patients with unique IC factor	90.9 (10/11)
Patient with two IC factors	9.1 (1/11)
Type of IC factors for cryptococcoses (*n* = 11 patients with 12 IC factors)	
Human immunodeficiency virus infection	54.5 (6/11)
Solid organ transplantation	27.3 (3/11)
Dyskeratosis congenita	9.1 (1/11)
Chemotherapy for colorectal cancer	9.1 (1/11)
Chemotherapy for T-cell prolymphocytic leukemia	9.1 (1/11)
Histoplasmoses (*n* = 10 patients)	
IC patients	60 (6/10)
Non-IC patients	20 (2/10)
IC status not known	20 (2/10)
Patients with unique IC factor	83.3 (5/6)
Patients with two IC factors	16.7 (1/6)
Type of IC factors for histoplasmoses (*n* = 6 patients with 7 IC factors)	
Human immunodeficiency virus infection	66.7 (4/6)
Diabetes	16.7 (1/6)
Chemothgerapy for lung cancer	16.7 (1/6)
Splenectomy	16.7 (1/6)
**Sample type and location** (27 FT), % (*n*/*N*)	
Biopsies	74.1 (20/27)
Bronchus/lung	40 (8/20)
Skin	30 (6/20)
Lymph node	20 (4/20)
Gastrointestinal tract	10 (2/20)
Surgical resections	25.9 (7/27)
Lung	85.7 (6/7)
Colon	14.3 (1/7)

FT: formalin-fixed paraffin-embedded tissues; IC: Immunocompromised; *N*: total number of FT; *n*: number of FT.

Concerning cryptococcoses (*n* = 16 FT from 13 patients; groups 1 and 2), mycological identification was not possible on fresh samples in the mycology laboratory in 6/13 (46.2%) patients; this was explained either by the absence of fresh tissue/CSF/blood/serum sent to the mycology laboratory in 4/6 (66.7%) patients or by negative cultures in 2/6 (33.3%) patients. For these six patients without mycological identification available on fresh sample, an integrated histomolecular diagnosis using panfungal PCR followed by Sanger sequencing and/or targeted MPS on FT led to the identification of *C. neoformans/gattii* in 5/6 (83.3%) FT tested. Overall, panfungal PCR followed by Sanger sequencing on FT was successful in 7/16 (43.8%) FT tested and panfungal PCR followed by targeted MPS in 12/14 (85.7%) FT. A statistically significant difference between both sequencings was observed in favor of targeted MPS (*P *= .041).

Concerning histoplasmoses (*n* = 11 FT from 10 patients; groups 1 and 2), the mycological identification was not possible on fresh samples in the mycology laboratory in 6/10 (60%) patients; this was explained by the absence of fresh tissue sent to the mycology laboratory in all 6/6 (100%) patients. For these six patients without mycological identification available on fresh tissue, an integrated histomolecular diagnosis using specific *H. capsulatum* PCR, and/or panfungal PCR followed by Sanger sequencing and/or targeted MPS on FT led to the identification of *H. capsulatum* var*. capsulatum* in 4/6 (66.7%) FT. Overall, specific *H. capsulatum* PCR was successful in 4/5 (80%) FT tested. Panfungal PCR followed by Sanger sequencing was successful for 4/10 (40%) patients on FT tested. Panfungal PCR followed by targeted MPS was successful in 3/5 (60%) FT tested. No significant difference was observed between the sensitivity of Sanger sequencing and targeted MPS (*P *= .24).

Clinical, laboratory, molecular, and sampling data are presented in Tables 1, 2, and Supplementary Table 1.

**Table 2.** Main laboratory and histopathological data.

FT #	Group	Final diagnosis	Results of culture and/or cryptococcal antigen	Specific *H. capsulatum* PCR on FT	Panfungal PCR + Sanger sequencing on FT	Panfungal PCR + targeted MPS on FT	Protozoa-like feature	Capsule or pseudo-capsule on the HES	Oval shape	Round shape	Dented-looking	Presence of pseudohyphae	Size	Alcian blue positivity
1*	1	Cryptococcosis	Positive hemoculture	NP	Positive PCR *C. neoformans/ gattii*	Positive PCR *C. neoformans/ gattii*	No	Clearly visible	No	Yes	No	No	Variable	Yes, intense
2*	1	Cryptococcosis	Positive hemoculture	NP	Positive PCR *C. neoformans/ gattii*	Positive PCR *C. neoformans/ gattii*	No	Clearly visible	Yes	Yes	Yes	No	Variable	Yes, intense
3*	1	Cryptococcosis	Positive hemoculture, Positive antigen (serum)	NP	Positive PCR *C. neoformans/ gattii*	NP	No	Clearly visible	Yes	Yes	Yes	No	Variable	Yes, intense
4	1	Cryptococcosis	NP	NP	Negative	Positive PCR *C. neoformans/ gattii*	No	Clearly visible	Yes	Yes	Yes	Yes	Variable	Yes, intense
5	1	Cryptococcosis	Positive antigen (serum)	NP	Positive PCR *C. neoformans/ gattii*	Positive PCR *C. neoformans/ gattii*	No	Clearly visible	No	Yes	Yes	No	Variable	Yes, intense
6	1	Cryptococcosis	Positive antigen (CSF)	NP	Negative	Negative	No	Clearly visible	No	Yes	No	No	Variable	Yes, intense
7	1	Cryptococcosis	Negative culture (BAL)	NP	Negative	Positive PCR *C. neoformans/ gattii*	No	Clearly visible	No	Yes	Yes	No	Variable	Yes, weak
8	1	Cryptococcosis	Positive culture (CSF), Positive antigen (serum)	NP	Positive PCR *C. neoformans/ gattii*	Positive PCR *C. neoformans/ gattii*	No	Clearly visible	Yes	Yes	Yes	Yes	Variable	Yes, intense
9	1	Cryptococcosis	Positive culture (tissue), Positive antigen (serum)	NP	Positive PCR *C. neoformans/ gattii*	Positive PCR *C. neoformans/ gattii*	No	Clearly visible	Yes	Yes	Yes	Yes	Variable	Yes, intense
10**	1	Cryptococcosis	Negative culture (tissue)	NP	Negative	Positive PCR *C. neoformans/ gattii*	No	Clearly visible	Yes	Yes	No	No	Variable	Yes, weak
11**	1	Cryptococcosis	Positive culture (tissue), Positive antigen (serum)	NP	Negative	Negative	No	Not visible	No	Yes	Yes	No	Variable	Yes, weak
12	1	Cryptococcosis	NP	NP	Negative	Positive PCR *C. neoformans/ gattii*	No	Not visible	No	Yes	No	No	Variable	Yes, weak
13	1	Cryptococcosis	Positive culture (tissue)	NP	Positive PCR *C. neoformans/ gattii*	Positive PCR *C. neoformans/ gattii*	No	Clearly visible	No	Yes	No	No	Variable	Yes, intense
14	1	Cryptococcosis	Negative cultures (tissue and CSP), Positive antigen (serum)	NP	Negative	Positive PCR *C. neoformans/ gattii*	No	Clearly visible	No	Yes	Yes	No	Variable	Yes, weak
15	1	Cryptococcosis	NP	NP	Negative	Positive PCR *C. neoformans/ gattii*	No	Clearly visible	No	Yes	Yes	No	Variable	Yes, intense
16	1	Histoplasmosis	NP	Positive	Negative	NP	No	Not visible	Yes	No	No	No	Homogenous	Absence (microcalcifications weakly positive)
17***	1	Histoplasmosis	Positive culture (tissue)	Positive	Positive PCR *H. capsultatum*	NP	No	Not visible	Yes	No	No	No	Homogenous	Absence
18***	1	Histoplasmosis	Positive culture (tissue)	Positive	Positive PCR *H. capsultatum*	NP	No	Not visible	Yes	No	No	No	Homogenous	Absence
19	1	Histoplasmosis	Negative culture (tissue)	Positive	Negative	NP	No	Not visible	Yes	No	No	No	Homogenous	Absence
20	1	Histoplasmosis	NP	NP	Positive PCR: *H.capsultatum*	NP	Yes	Not visible	Yes	No	No	No	Homogenous	Absence
21	1	Histoplasmosis	NP	NP	Negative	Positive PCR: *H. capsultatum*	No	Not visible	Yes	No	No	No	Homogenous	Absence
22	1	Histoplasmosis	Positive culture (tissue)	NP	Negative	Positive PCR: *H. capsultatum*	No	Not visible	Yes	Yes	No	No	Homogenous	Absence
23	1	Histoplasmosis	Negative culture (tissue)	NP	Negative	Negative	No	Pseudocapsule	Yes	No	No	No	Homogenous	Absence
24	1	Histoplasmosis	NP	NP	Positive PCR: *H. capsultatum*	Positive PCR: *H. capsultatum*	Yes	Not visible	Yes	No	No	No	Homogenous	Absence
25	2	Cryptococcosis	NP	NP	Negative	NP	No	Clearly visible	No	Yes	Yes	No	Variable	Yes, weak
26	2	Histoplasmosis	NP	Negative	Negative	Negative	No	No	Yes	No	No	No	Homogenous	Absence
27	2	Histoplasmosis	NP	NP	NP	NP	Yes	No	Yes	No	No	No	Homogenous	Absence

BAL: bronchoalveolar lavage; CSF: cerebrospinal fluid; FT: formalin-fixed, paraffin-embedded tissues; MPS: massive parallel sequencing; NP: not performed; PCR: polymerase chain reaction. *Samples belonging to the same patient; **samples belonging to the same patient; ***samples belonging to the same patient.

### Histopathological analysis of group 1 FT (with mycological identification; *n* = 24 FT from 20 patients) and comparison between cryptococcosis vs. histoplasmosis

**Figure 2.**
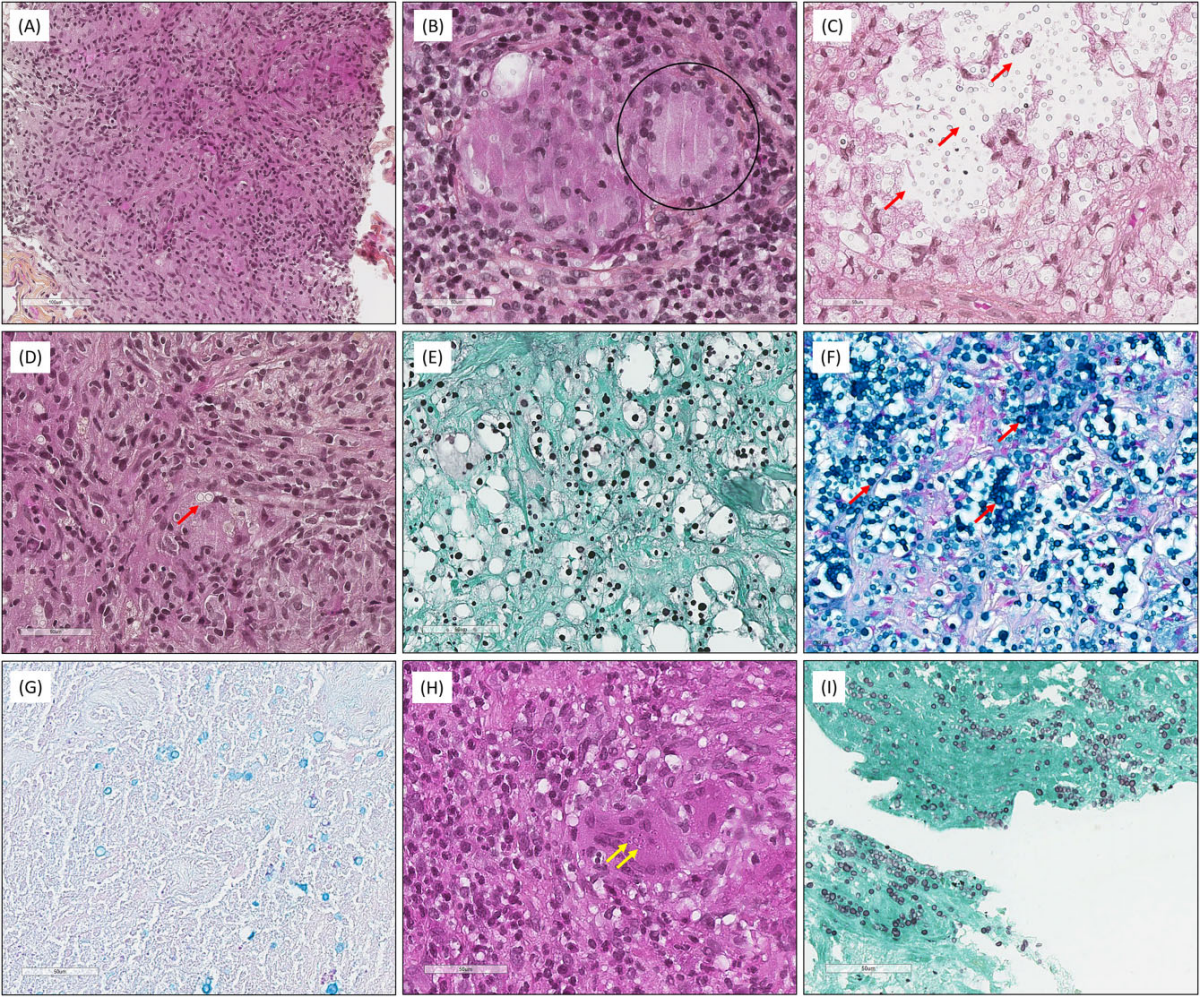
Classical histopathological features observed in cryptococcosis and histoplasmosis. (A) (Hematoxylin–eosin–saffron [HES], ×200), granulomatous inflammation. (B) (HES, ×400), giant-cells (black circle). (C) (HES, ×400), capsule of *Cryptococcus neoformans* clearly visible (red arrows). (D) (HES, ×400), inconspicuous capsule of *C. neoformans* (red arrow). (E) (Grocott, ×400), round yeast of variable/heterogenous size (*C. neoformans*). (F) (Alcian blue, ×400), *C. neoformans* capsule intense positivity (red arrows). (G) (Alcian blue, ×400), *C. neoformans* capsule weak positivity (red arrows). (H) (HES, ×400), pseudo-capsule of *Histoplasma capsulatum* var*. capsulatum* (yellow arrows) on a giant cell. (I) (Grocott, ×400), oval yeasts of homogenous size (*H. capsulatum* var*. capsulatum*).

**Figure 3.**
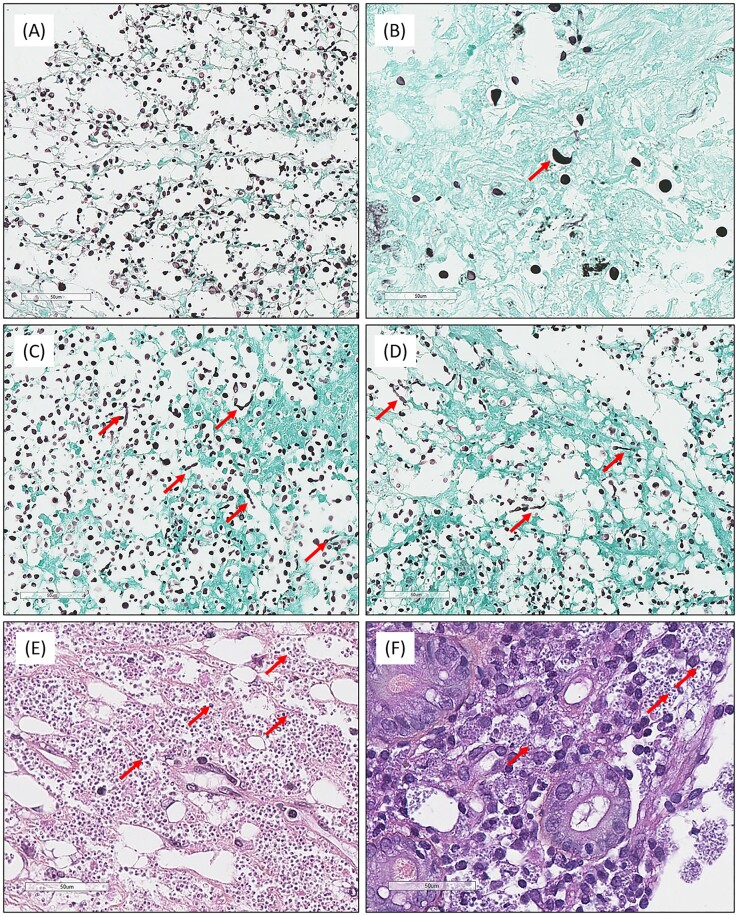
Atypical histopathological features were observed in the present study. (A) (Grocott, ×400) and (B) (Grocott, ×500), dented-looking yeast (*Cryptococcus neoformans*) (red arrows). (C) (Grocott, ×400) and (D) (Grocott, ×400), Pseudohyphae formation (*C. neoformans*; red arrows). (E) (Hematoxylin–eosin–saffron [HES], ×400) and (F) (HES, ×400). “Protozoa-like” features (*Histoplasma capsulatum* var*. capsulatum*, red arrows).

**Figure 4.**
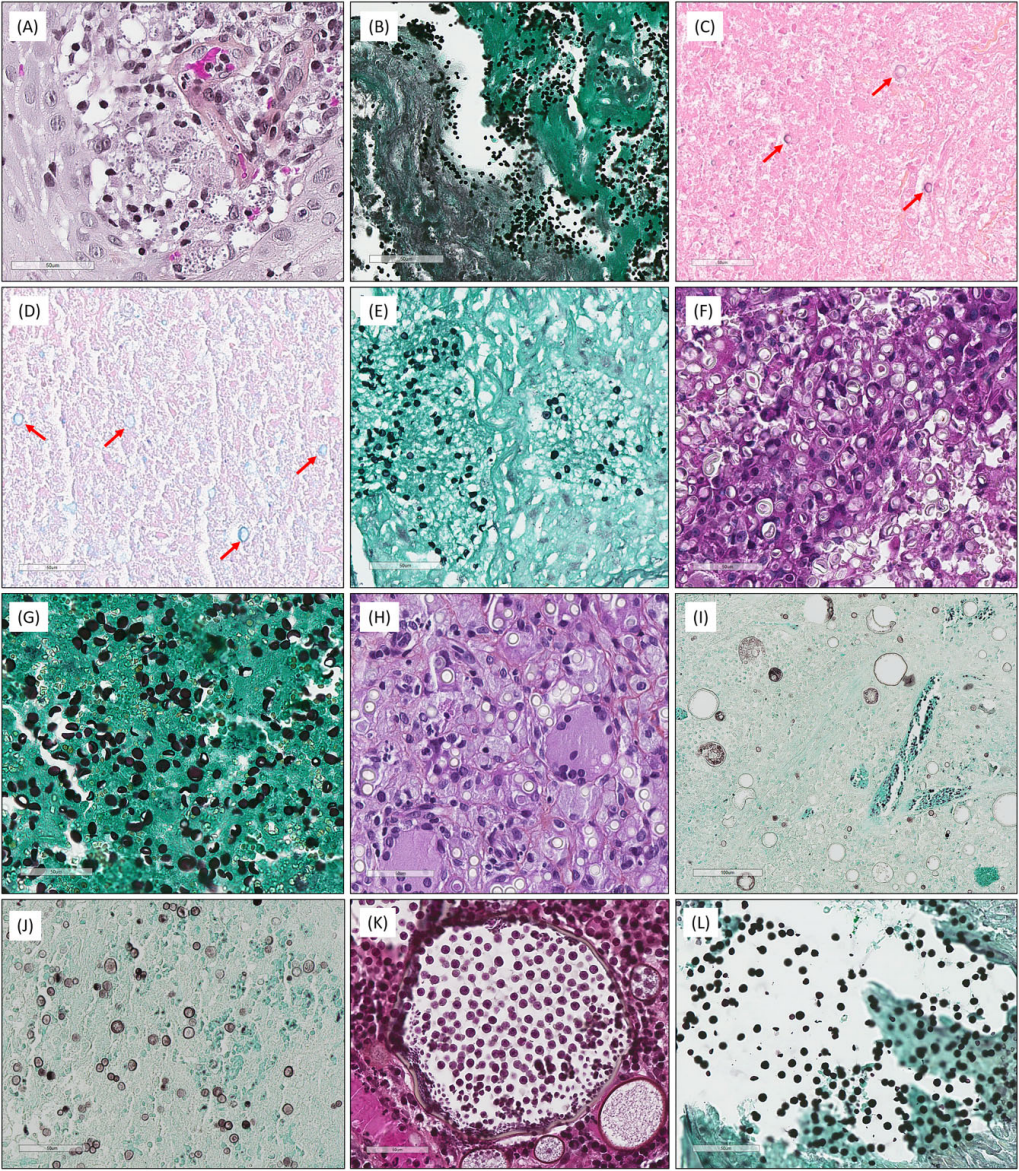
Histopathological differential diagnoses of *Cryptococcus neoformans/gattii* and *Histoplasma capsulatum* var*. capsulatum*. (A) (Hematoxylin–eosin–saffron [HES], ×400), *Leishmania* amastigotes of a cutaneous leishmaniasis. (B) (Grocott, ×400), *Candida glabrata* placental membrane infection. (C) (HES, ×400) and (D) (Alcian blue, ×400), microcalcifications in necrosis, sometimes slightly stained (red arrows). (E) (Grocott, ×400), *Pneumocystis jirovecii* cysts. (F) (HES, ×400) and (G) (Grocott, ×400), *H. capsulatum* var*. duboisii* cutaneous infection. (H) (HES, ×400), cutaneous blastomycosis (*Blastomyces dermatitidis*). (I) (Grocott, ×200) spherule of *Coccidioides immitis* and its endospores spilled into surrounding tissues (J; Grocott, ×400). (K) (HES, ×400) and (L) (Grocott, ×400). Sporangia of *Rhinosporidium* spp. with endospores sometimes spilled into surrounding tissues.

The proportions of histopathological features observed for cryptococcosis (*n* = 15 FT) and histoplasmosis (*n* = 9 FT) of group 1 are presented and compared in Tables 2 and 3, and fully detailed in Supplementary Tables 2 and 3. For cryptococcoses, the capsule was clearly visible in 13/15 (86.7%) FT and not visible or inconspicuous in 2/15 (13.3%) on the HES-stained slide. The capsule was weakly (5/15, 33.3%) or intensely (10/15, 66.7%) stained on the Alcian blue-stained slide. Conversely, Alcian blue staining was always negative (0/9, 0%) for histoplasmoses. However, in 1/9 (9.1%) FT, microcalcifications were weakly stained. Figures 2 and 3 illustrate the histopathological features observed in the present study. The main histopathological differential diagnoses are presented in Fig. 4.

**Table 3.** Histopathological results of group 1 FT.

Histopathological features	Cryptococcosis, *n*/*N* (%)	Histoplasmosis, *n*/*N* (%)	*P* values*
Granulomatous inflammation	12/15 (80)	8/9 (88.8)	1
Giant cells	7/15 (46.7)	2/9 (22.2)	.3891
Necrosis	6/15 (40)	5/9 (55.5)	.6752
Caseous necrosis	5/15 (33.3)	4/9 (44.4)	.6785
Visible capsule or pseudocapsule	13/15 (86.7)	1/9 (11.1)	**.0005**
Extracellular location**	13/15 (86.7)	5/9 (55.5)	.1501
Intracellular location	12/12 (100)***	7/8 (87.5)***	1
‘Protozoa-like’ features	0/15 (0)	2/9 (22.2)	.1304
Round yeasts	15/15 (100)	1/9 (11.1)	**<.0001**
Oval yeasts	6/15 (40)	9/9 (100)	**.0068**
Dented-looking yeasts	10/15 (66.7)	0/9 (0)	**.002**
Pseudohyphae	3/15 (20)	0/9 (0)	.2663
Yeasts of variable size	15/15 (100)	0/9 (0)	**<.0001**
Yeasts of homogenous size	0/15 (100)	9/9 (100)	**<.0001**
Positive Alcian blue staining	15/15 (100)	0/9 (0)	**<.0001**

*In bold type, statistically significant results.

**Either extracellular yeasts within necrosis or extracellular yeasts outside necrosis area and between inflammatory cells.

***After exclusion of FT without tissue or human cells for which the intracellular location could not be assessed.

FT: formalin-fixed paraffin-embedded tissues; *N*: total number of FT; *n*: number of FT.

### Histopathological analysis of group 2 FT (without mycological identification; *n* = 3 FT from 3 patients)

Concerning the histopathologically suspected cryptococcosis of group 2 (*n* = 1 FT), all histopathological features of interest previously identified in group 1 were concordant with the diagnosis of cryptococcosis. On the HES-stained slide, the capsule was clearly visible in 1/1 FT. On the Grocott-stained slides, the shape of the yeasts was round (1/1) and combined with dented-looking (1/1) yeasts, without oval yeast (0/1). The yeast size was variable/heterogenous (1/1). The capsule was weakly stained by Alcian blue in 1/1 FT.

Concerning the histopathologically suspected histoplasmosis of group 2 (*n* = 2 FT), all histopathological features of interest previously identified in group 1 were concordant with the diagnosis of histoplasmosis. On the HES-stained slide, no capsule was visible (0/2). On the Grocott-stained slides, the shape of the yeasts was always oval (2/2), without round or dented-looking yeast (0/2). The yeast size was always homogeneous (2/2). Alcian blue staining was always negative (0/2).

## Discussion

In the present study, we aimed to estimate the frequency of atypical histopathological features observed in *C. neoformans* and *H. capsulatum* var*. capsulatum* infections to investigate whether these features could be useful to differentiate both mycoses on FT. Indeed, the morphological differential diagnosis between these mycoses is sometimes difficult in practice. Thus, we also assessed the relevance of an integrated histomolecular diagnosis on FT for diagnostic accuracy and safety. We found that dented-looking yeasts were an atypical histopathological feature significantly associated with *C. neoformans/gattii* infections. Although the presence of pseudohyphae and the “protozoa-like” feature were more present in *C. neoformans/gattii* and *H. capsulatum* var. *capsulatum* infections, respectively, this was only observed as a trend without statistical significance. When no mycological identification was available on fresh tissue, an integrated histomolecular diagnosis on FT led to a mycological identification in three-quarters of the patients, improving fungal identification and patient care.

This last point highlights the necessity to develop fungal integrated histomolecular diagnosis on FT, especially in cases of clinically misleading infections.^8,9^ Indeed, in the present study, the absence of mycological identification was mainly explained by the absence of a fresh sample sent to the mycology laboratory because of a pseudotumor presentation. These tricky presentations may be explained by the inflammatory reaction, including an exuberant granulomatous or myofibroblastic response, and the immune status of the patients.^1,8,9^ This strengthens the role of the pathologists in the morphological diagnosis of cryptococcosis and histoplasmosis, especially when clinical and radiological data are deceptive.

However, histopathological diagnosis may also be difficult, as shown by the atypical features observed in the present study. For *H. capsulatum* var*. capsulatum*, the “protozoa-like” feature on the HES, observed in nearly a quarter of FT, may be misleading with *Leishmania* spp.,^15^ especially in cutaneous samples or in osteo-medullary biopsies. Moreover, the inflammatory reaction is granulomatous in both infections, as both pathogens are located in the cytoplasm of histiocytes.^15^ However, although the distinction on the HES-stained slide may be difficult, the Grocott-stained slide solves this diagnostic issue (i.e., positive stain for *H. capsulatum* var*. capsulatum* and negative stain for *Leishmania* spp.).^15^ Because *H. capsulatum* var*. capsulatum* is not reported to produce pseudohyphae, as also observed herein, *Candida glabrata* may be another differential diagnosis. However, this yeast is more variable in size and not exclusively of intracellular location compared to *H. capsulatum* var*. capsulatum*. Moreover, the inflammatory reaction due to *C. glabrata* infection is mainly composed of neutrophils and not histiocytes,^1^ highlighting the importance of performing a morphological analysis of tissue inflammation. Finally, the differential diagnosis between *H. capsulatum* var*. capsulatum* and *C. neoformans/gattii* may be challenging in practice, while the treatment of both mycoses differs.^16,17^ Indeed, for disseminated cryptococcosis, the recommended induction therapy is liposomal amphotericin B at 3 mg/kg daily and flucytosine at 25 mg/kg four times a day for 2 weeks, then consolidation therapy with fluconazole 400–800 mg for 8 weeks, and then maintenance therapy with fluconazole 200 mg daily for 12 months or until immune restoration.^18^ In contrast, the recommended treatment for disseminated histoplasmosis consists of an induction therapy with liposomal amphotericin B at 3 mg/kg daily and then itraconazole (200 mg twice daily) for at least 1 year.^19^ The diagnostic difficulties are highlighted by the discrepancies between the initial and expert pathologists herein, mainly represented by the confusion between both mycoses. This is especially true in the case of an absence of capsule on the HES-stained slide, observed in approximately 15% of the cryptococcoses in the present study. Moreover, initial intracellular forms of cryptococci have been reported as smaller than classical cryptococci, with a thinner capsule,^20^ complicating the distinction with intracellular *H. capsulatum* var*. capsulatum*. Indeed, the capsule size of *C. neoformans/gattii* may depend on the importance of the immunosuppression (i.e., well-developed capsule in strongly immunocompromised patients and inconspicuous capsule in weakly immunocompromised patients).^3^ Similarly, it has been hypothesized that the presence of pseudohyphae in cryptococcosis may also be rather observed in severe immunosuppression,^21^ whereas one patient herein was apparently non-IC. The presence of pseudohyphae in the case of *C. neoformans/gattii* infection should raise the differential diagnosis of *Candida* spp.^1^; however, the tissue inflammatory reaction is different (granulomatous for cryptococcosis vs. suppurative for candidiasis).^1^ For *C. neoformans/gattii*, both criteria (i.e., variable size of capsule, pseudohyphae) suggest that the immune status may not only influence the clinical outcomes,^9^ but also the morphology of the fungi within the tissues. This underlines the importance of being aware of the clinical data at the time of the histopathological analysis. In addition, dented-looking yeasts, observed in >65% of cryptococcoses herein, may be tricky but were not seen in histoplasmoses. This could be a helpful criterion to differentiate both mycoses on FT, especially when mycological confirmation on fresh tissue or FT is not possible, which represented 11.1% of the FT included herein (i.e., group 2), reinforcing the role of histopathological analysis. However, in lung samples, dented-looking yeasts should not be confused with irregular microcalcifications within necrosis (which can sometimes be slightly stained with Alcian blue, as observed in one patient herein) or the “deflated ball” shape of *Pneumocystis jirovecii*. Other large round/oval yeasts must be ruled out, such as *H. capsulatum* var. *duboisii* and *Blastomyces dermatitidis*, especially since the latter can also be highlighted with Alcian blue.^1,3^ Similarly, on biopsy samples, endospores of *Coccidioides immitis* or *Rhinosporidium* spp. spilled into surrounding tissues may mimic *C. neoformans*; in these cases, complete spherules and sporangia should be searched for on tissue sections.^1,3^

All these morphological pitfalls and differential diagnoses on FT underline the crucial role of a slide review by an infectious disease pathologist. Herein, this review led to the modification of the diagnosis for a quarter of the patients, which is comparable with data previously reported (22.9%).^2^ However, the number of infectious disease pathologists is continuously decreasing,^22,23^ which seems paradoxical given the increasing number of immunocompromised patients and fungal infections worldwide.^24^ The clinical relevance of integrated histomolecular diagnosis in the field of infectious disease pathology has been demonstrated several times.^2,22^,^25–27^ Thus, innovative molecular techniques must be developed on FT to improve fungal identification and thus patient care, especially when no fresh sample is sent to the mycology laboratory. Herein, the majority of patients without mycological identification on fresh tissue benefited from an integrated histomolecular diagnosis that led to fungal detection and identification. Moreover, for cryptococcosis, the sensitivity of targeted MPS was higher than that of Sanger sequencing on FT, confirming previous results from our group.^2^

The present study has some limitations. All molecular techniques could not always be performed for each FT because of the limited quantity of tissue sample. Indeed, almost three-quarters of samples were biopsy samples, which are representative of samples available in routine practice. Moreover, in group 2, the fungal density on the slides was low. In addition, the study was conducted using consultation cases, which means that the tissue preparation parameters (cold ischemia time, formalin fixation time, and duration) were not controlled by our center. This may explain the absence of identification for three patients (group 2). Thus, we tried to estimate and compare the proportion of atypical features between *C. neoformans* and *H. capsulatum* var*. capsulatum* to investigate if these criteria could be useful to differentiate both mycoses. However, the proportion of atypical features could be increased in the present study due to the expertise activity bias (i.e., “typical” infections may not have been referred for review). Finally, these types of histomolecular diagnostic methods cannot be widely used, particularly in resource-limited countries where these fungal infections are common.

To conclude, atypical histopathological features of *C. neoformans/gattii* and *H. capsulatum* var*. capsulatum* infections may be tricky. However, the presence of dented-looking yeasts could suggest cryptococcosis rather than histoplasmosis. In case of atypical features and/or absence of mycological identification on fresh samples, an integrated histomolecular diagnosis is essential for optimal patient care.

## Supplementary Material

myae126_Supplemental_File
