# Supplementary material for: Cryptococcus neoformans/gattii and Histoplasma capsulatum var. capsulatum infections on tissue sections: Diagnostic pitfalls and relevance of an integrated histomolecular diagnosis
Source: Med Mycol. 2024 Dec 28;63(1):myae126. doi: 10.1093/mmy/myae126 (PMC11735191; doi:10.1093/mmy/myae126)
Supplement: myae126_Supplemental_File [file myae126_supplemental_file.docx]

**Title**: *Cryptococcus neoformans/gattii* and *Histoplasma capsulatum* var. *capsulatum* infections on tissue sections: diagnostic pitfalls and relevance of an integrated histomolecular diagnosis

Supplementary information

**Supplemental Table S1**. Clinical and laboratory data, results from molecular tests on FT

**Supplemental Table S2**. Review of the slides and histopathological features analyzed on the HES-stained slides

**Supplemental Table S3**. Histopathological features analyzed on the Grocott-stained slides and Alcian blue-stained slide

| FT # | Group | Age at diagnosis | Sex | Immune status | Sample type | Location | Final diagnosis | Results of direct examination | Results and type of culture | Panfungal PCR + Sanger sequencing on fresh tissue |  | Cryptococcal antigen | Specific *Histoplasma capsulatum* PCR on FT | Panfungal PCR + Sanger sequencing on FT | Panfungal PCR + targeted-MPS on FT |
| --- | --- | --- | --- | --- | --- | --- | --- | --- | --- | --- | --- | --- | --- | --- | --- |
| 1* | 1 | 42 | M | IC (HIV) | Biopsy | Lymph node | Cryptococcosis | NP | Positive hemoculture | NP |  | NP | NP | Positive PCR (MITS2A/2B primers): *C. neoformans/gattii* | Positive PCR (MITS2A/2B primers): *C. neoformans/gattii* |
| 2* | 1 | 42 | M | IC (HIV) | Biopsy | Skin | Cryptococcosis | NP | Positive hemoculture | NP |  | NP | NP | Positive PCR (MITS2A/2B primers): *C. neoformans/gattii* | Positive PCR (MITS2A/2B primers): *C. neoformans/gattii* |
| 3* | 1 | 44 | M | IC (HIV) | Biopsy | Skin | Cryptococcosis | NP | Positive hemoculture | NP |  | Positive (serum) | NP | Positive PCR (MITS2A/2B primers): *C. neoformans/gattii* | NP |
| 4 | 1 | 59 | M | NA | Biopsy | Lung | Cryptococcosis | NP | NP | NP |  | NP | NP | Negative (MITS2A/B and ITS3/4 primers) | Positive PCR (MITS2A/2B primers): *C. neoformans/gattii* |
| 5 | 1 | 46 | F | IC (HIV) | Biopsy | Lymph node | Cryptococcosis | NP | NP | NP |  | Positive (serum) | NP | Positive PCR (MITS2A/2B primers): *C. neoformans/gattii* | Positive PCR (MITS2A/2B primers): *C. neoformans/gattii* |
| 6 | 1 | 54 | F | Kidney transplant | Biopsy | Skin | Cryptococcosis | NP | NP | NP |  | Positive (CSF) | NP | Negative (MITS2A/B and ITS3/4 primers) | Negative (MITS2A/B and ITS3/4 primers) |
| 7 | 1 | 69 | M | IC (HIV) | Biopsy | Lung | Cryptococcosis | NP | Negative (BAL) | NP |  | NP | NP | Negative (MITS2A/B and ITS3/4 primers) | Positive PCR (MITS2A/2B primers): *C. neoformans/gattii* |
| 8 | 1 | 56 | M | IC (HIV) + liver transplant | Biopsy | Lung | Cryptococcosis | NP | Positive (CSF) | NP |  | Positive (serum) | NP | Positive PCR (MITS2A/2B primers): *C. neoformans/gattii* | Positive PCR (MITS2A/2B primers): *C. neoformans/gattii* |
| 9 | 1 | 80 | M | Prolymphocytic T-cell leukemia | Biopsy | Skin | Cryptococcosis | NP | Positive (tissue) | NP |  | Positive (serum) | NP | Positive PCR (MITS2A/2B primers): *C. neoformans/gattii* | Positive PCR (MITS2A/2B primers): *C. neoformans/gattii* |
| 10** | 1 | 59 | M | Kidney transplant | Surgical specimen | Lung | Cryptococcosis | Positive (CSP) | Negative (tissue) | NP |  | NP | NP | Negative (MITS2A/B and ITS3/4 primers) | Positive PCR (MITS2A/2B primers): *C. neoformans/gattii* |
| 11** | 1 | 59 | M | Kidney transplant | Surgical specimen | Lung | Cryptococcosis | Positive (CSP) | Positive (tissue) | NP |  | Positive (serum) | NP | Negative (MITS2A/B and ITS3/4 primers) | Negative (MITS2A/B and ITS3/4 primers) |
| 12 | 1 | 42 | M | Dyskeratosis congenita | Surgical specimen | Lung | Cryptococcosis | NP | NP | NP |  | NP | NP | Negative (MITS2A/B and ITS3/4 primers) | Positive PCR (MITS2A/2B primers): *C. neoformans/gattii* |
| 13 | 1 | 48 | M | IC (HIV) | Biopsy | Lung | Cryptococcosis | NP | Positive (tissue) | NP |  | NP | NP | Positive PCR (MITS2A/2B primers): *C. neoformans/gattii* | Positive PCR (MITS2A/2B primers): *C. neoformans/gattii* |
| 14 | 1 | 52 | F | IC (HIV) | Biopsy | Stomac | Cryptococcosis | Positive  (CSP) | Negative (tissue and CSP) | NP |  | Positive (serum) | NP | Negative (MITS2A/B and ITS3/4 primers) | Positive PCR (MITS2A/2B primers): *C. neoformans/gattii* |
| 15 | 1 | 69 | F | Chemotherapy for colorectal cancer | Surgical specimen | Lung | Cryptococcosis | NP | NP | NP |  | NP | NP | Negative (MITS2A/B primers) | Positive PCR (MITS2A/2B primers): *C. neoformans/gattii* |
| 16 | 1 | 65 | M | Chemotherapy for lung cancer | Surgical specimen | Lung | Histoplasmosis | NP | NP | NP |  | NP | Positive | Negative (MITS2A/B and ITS3/4 primers) | NP |
| 17*** | 1 | 46 | F | IC (HIV) | Biopsy | Skin | Histoplasmosis | Positive (tissue) | Positive (tissue) | Positive PCR (MITS2A/2B primers) : *H. capsultatum* |  | NP | Positive | Positive PCR (MITS2A/2B primers): *H. capsultatum* | NP |
| 18*** | 1 | 46 | F | IC (HIV) | Biopsy | Skin | Histoplasmosis | Positive (tissue) | Positive (tissue) | Positive PCR (MITS2A/2B primers) : *H. capsultatum* |  | NP | Positive | Positive PCR (MITS2A/2B primers): *H. capsultatum* | NP |
| 19 | 1 | 39 | F | IC (HIV) | Biopsy | Lymph node | Histoplasmosis | NP | Negative (tissue) | Positive PCR (MITS2A/2B primers) : *H. capsultatum* |  | NP | Positive | Negative (MITS2A/B and ITS3/4 primers) | NP |
| 20 | 1 | 72 | F | IC (HIV) | Biopsy | Colon | Histoplasmosis | NP | NP | NP |  | NP | NP | Positive PCR (MITS2A/2B and ITS3/4 primers): *H. capsultatum* | NP |
| 21 | 1 | 48 | F | NA | Surgical specimen | Lung | Histoplasmosis | NP | NP | NP |  | NP | NP | Negative (MITS2A/B and ITS3/4 primers) | Positive PCR (ITS3/4 primers): *H. capsultatum* |
| 22 | 1 | 71 | M | Non-IC | Biopsy | Bronchus | Histoplasmosis | NP | Positive (tissue) | NP |  | NP | NP | Negative (MITS2A/B and ITS3/4 primers) | Positive PCR (MITS2A/2B and ITS3/4 primers): *H. capsultatum* |
| 23 | 1 | 70 | M | Diabetes + splenectomy | Biopsy | Lung | Histoplasmosis | Positive (BAL) | Negative (tissue) | Positive PCR (MITS2A/2B primers) : *H. capsultatum* |  | NP | NP | Negative (MITS2A/B and ITS3/4 primers) | Negative (MITS2A/B and ITS3/4 primers) |
| 24 | 1 | 53 | F | NA | Surgical specimen | Colon | Histoplasmosis | NP | NP | NP |  | NP | NP | Positive PCR (MITS2A/2B and ITS3/4 primers): *H. capsultatum* | Positive PCR (MITS2A/2B and ITS3/4 primers): *H. capsultatum* |
| 25 | 2 | 44 | M | Non-IC | Biopsy | Lung | Cryptococcosis | NP | NP | NP |  | NP | NP | Negative (MITS2A/B and ITS3/4 primers) | NP |
| 26 | 2 | 53 | F | Non-IC | Biopsy | Lung | Histoplasmosis | NP | NP | NP |  | NP | Negative | Negative (MITS2A/B and ITS3/4 primers) | Negative (MITS2A/B and ITS3/4 primers) |
| 27 | 2 | 35 | M | IC (HIV) | Biopsy | Lymph node | Histoplasmosis | NP | NP | NP |  | NP | NP | NP | NP |

**Supplementary Table S1: Clinical and laboratory data, results from molecular tests on FT**

BAL: Bronchoalveolar lavage; CSP: Cerebrospinal fluid; IC: Immunocompromised; F: Female; FT: Formalin-fixed, paraffin-embedded tissues; HIV: Human immunodeficiency virus; M: Male; MPS: Massive parallel sequencing; NP: Not performed; PCR: Polymerase chain reaction. *Samples belonging to the same patient; **Samples belonging to the same patient; ***Samples belonging to the same patient.

| FT # | Group | First diagnosis (non-expert) | Final diagnosis (after expert review of the slides) | Diagnosis confirmation | Diagnosis modification | Granulomatous inflammation | Giant cells | Presence of necrosis | Presence of caseous necrosis | Capsule or pseudo-capsule on the HES | “Protozoa-like” feature | Intra-cellular location | Extra-cellular location |
| --- | --- | --- | --- | --- | --- | --- | --- | --- | --- | --- | --- | --- | --- |
| 1 | 1 | Cryptococcosis | Cryptococcosis | Yes | - | Yes | Yes | No | No | Clearly visible | No | Yes | Yes |
| 2 | 1 | Cryptococcosis | Cryptococcosis | Yes | - | Yes | Yes | No | No | Clearly visible | No | Yes | No |
| 3 | 1 | Cryptococcosis | Cryptococcosis | Yes | - | Yes | No | No | No | Clearly visible | No | Yes | No |
| 4 | 1 | Cryptococcosis | Cryptococcosis | Yes | - | Necrosis only | Necrosis only | Necrosis only | No | Clearly visible | No | No, necrosis only | Yes (within the necrosis) |
| 5 | 1 | Cryptococcosis | Cryptococcosis | Yes | - | Yes | Yes | No | No | Clearly visible | No | Yes | Yes |
| 6 | 1 | Cryptococcosis | Cryptococcosis | Yes | - | Yes | No | No | No | Clearly visible | No | Yes | Yes |
| 7 | 1 | Cryptococcosis | Cryptococcosis | Yes | - | Yes | Yes | No | No | Clearly visible | No | Yes | Yes |
| 8 | 1 | Cryptococcosis | Cryptococcosis | Yes | - | Necrosis only | Necrosis only | Necrosis only | Yes | Clearly visible | No | No, necrosis only | Yes (within the necrosis) |
| 9 | 1 | Cryptococcosis | Cryptococcosis | Yes | - | Yes | No | No | No | Clearly visible | No | Yes | Yes |
| 10 | 1 | Cryptococcosis | Cryptococcosis | Yes | - | Yes | No | Yes | Yes | Clearly visible | No | Yes | Yes |
| 11 | 1 | Cryptococcosis | Cryptococcosis | Yes | - | Necrosis only | Necrosis only | Necrosis only | Yes | Not visible | No | No, necrosis only | Yes (within the necrosis) |
| 12 | 1 | Histoplasmosis | Cryptococcosis | - | Yes | Yes | Yes | Yes | Yes | Not visible | No | Yes | Yes |
| 13 | 1 | Cryptococcosis | Cryptococcosis | Yes | - | Yes | Yes | No | No | Clearly visible | No | Yes | Yes |
| 14 | 1 | Cryptococcosis | Cryptococcosis | Yes | - | Yes | No | No | No | Clearly visible | No | Yes | Yes |
| 15 | 1 | Cryptococcosis | Cryptococcosis | Yes | - | Yes | Yes | Yes | Yes | Clearly visible | No | Yes | Yes |
| 16 | 1 | Cryptococcosis | Histoplasmosis | - | Yes | Yes | No | Yes | Yes | Not visible | No | No | Yes (within the necrosis) |
| 17 | 1 | Yeasts | Histoplasmosis | - | Yes | Yes | No | No | No | Not visible | No | Yes | No |
| 18 | 1 | Histoplasmosis | Histoplasmosis | Yes | - | Yes | No | No | No | Not visible | No | Yes | No |
| 19 | 1 | Histoplasmosis | Histoplasmosis | Yes | - | Yes | No | No | No | Not visible | No | Yes | No |
| 20 | 1 | Fungi | Histoplasmosis | - | Yes | Yes | No | Yes | No | Not visible | Yes | Yes | Yes (within the necrosis) |
| 21 | 1 | Spores | Histoplasmosis | - | Yes | Yes | Yes | Yes | Yes | Not visible | No | Yes | Yes (within the necrosis) |
| 22 | 1 | Yeasts | Histoplasmosis | - | Yes | Yes | No | Yes | Yes | Not visible | No | Yes | Yes (within the necrosis) |
| 23 | 1 | Histoplasmosis | Histoplasmosis | Yes | - | Yes | Yes | No | No | Pseudocapsule | No | Yes | No |
| 24 | 1 | Histoplasmosis | Histoplasmosis | Yes | - | Necrosis only | Necrosis only | Necrosis only | Yes | Not visible | Yes | No, necrosis only | Yes (within the necrosis) |
| 25 | 2 | Cryptococcosis | Cryptococcosis | Yes | - | Yes | Yes | No | No | Clearly visible | No | Yes | Yes |
| 26 | 2 | Histoplasmosis | Histoplasmosis | Yes | - | Necrosis only | Necrosis only | Necrosis only | Necrosis only | No | No | No, necrosis only | Yes (within the necrosis) |
| 27 | 2 | Crycptococcosis or Histoplasmosis | Histoplasmosis | - | Yes | Yes | No | Yes | Yes | No | Yes | Yes | Yes (within the necrosis) |

**Supplementary Table S2: Review of the slides and histopathological features analyzed on the HES-stained slides.**

HES: Hematoxylin-eosin-saffron.

| FT # | Group | Final diagnosis (after expert review of the slides) | Oval shape | Round shape | Dented-looking | Presence of pseudohyphae | Homogeneous or variable size | Alcian blue positivity (absence, weak, intense) |
| --- | --- | --- | --- | --- | --- | --- | --- | --- |
| 1 | 1 | Cryptococcosis | No | Yes | No | No | Variable | Yes, intense |
| 2 | 1 | Cryptococcosis | Yes | Yes | Yes | No | Variable | Yes, intense |
| 3 | 1 | Cryptococcosis | Yes | Yes | Yes | No | Variable | Yes, intense |
| 4 | 1 | Cryptococcosis | Yes | Yes | Yes | Yes | Variable | Yes, intense |
| 5 | 1 | Cryptococcosis | No | Yes | Yes | No | Variable | Yes, intense |
| 6 | 1 | Cryptococcosis | No | Yes | No | No | Variable | Yes, intense |
| 7 | 1 | Cryptococcosis | No | Yes | Yes | No | Variable | Yes, weak |
| 8 | 1 | Cryptococcosis | Yes | Yes | Yes | Yes | Variable | Yes, intense |
| 9 | 1 | Cryptococcosis | Yes | Yes | Yes | Yes | Variable | Yes, intense |
| 10 | 1 | Cryptococcosis | Yes | Yes | No | No | Variable | Yes, weak |
| 11 | 1 | Cryptococcosis | No | Yes | Yes | No | Variable | Yes, weak |
| 12 | 1 | Cryptococcosis | No | Yes | No | No | Variable | Yes, weak |
| 13 | 1 | Cryptococcosis | No | Yes | No | No | Variable | Yes, intense |
| 14 | 1 | Cryptococcosis | No | Yes | Yes | No | Variable | Yes, weak |
| 15 | 1 | Cryptococcosis | No | Yes | Yes | No | Variable | Yes, intense |
| 16 | 1 | Histoplasmosis | Yes | No | No | No | Homogenous | Absence (microcalcifications weakly positive) |
| 17 | 1 | Histoplasmosis | Yes | No | No | No | Homogenous | Absence |
| 18 | 1 | Histoplasmosis | Yes | No | No | No | Homogenous | Absence |
| 19 | 1 | Histoplasmosis | Yes | No | No | No | Homogenous | Absence |
| 20 | 1 | Histoplasmosis | Yes | No | No | No | Homogenous | Absence |
| 21 | 1 | Histoplasmosis | Yes | No | No | No | Homogenous | Absence |
| 22 | 1 | Histoplasmosis | Yes | Yes | No | No | Homogenous | Absence |
| 23 | 1 | Histoplasmosis | Yes | No | No | No | Homogenous | Absence |
| 24 | 1 | Histoplasmosis | Yes | No | No | No | Homogenous | Absence |
| 25 | 2 | Cryptococcosis | No | Yes | Yes | No | Variable | Yes, weak |
| 26 | 2 | Histoplasmosis | Yes | No | No | No | Homogenous | Absence |
| 27 | 2 | Histoplasmosis | Yes | No | No | No | Homogenous | Absence |
|  |  |  |  |  |  |  |  |  |

**Supplementary Table S3: Histopathological features analyzed on the Grocott-stained slides and Alcian blue-stained slides**

HES: Hematoxylin-eosin-saffron.
